# Management of subjects with type 2 diabetes hospitalized in internal medicine units: a cluster-randomized, multicenter study before and after an educational program

**DOI:** 10.1007/s11739-025-04238-1

**Published:** 2026-03-13

**Authors:** Antonio Ceriello, Andrea Fontanella, Tiziana Marcella Attardo, Giampietro Beltramello, Aldo Fierro, Francesco Prattichizzo, Valeria Pellegrini, Maria Serena Fiore, Ernesto De Menis, Ada Maffettone, Luigi Magnani, Ruggero Pastorelli, Francesco Ventrella, Massimo Rondana, Mauro Maurantonio, Mauro Scanferlato, Francesco Finizola, Maria Antonia Salvia, Riccardo Nevola, Giorgia Prampolini, Marco Laccetti, Franco Mastroianni, Fabio Cartabellotta, Elisa Zagarrì, Francesco Dentali, Dario Manfellotto, FADOI-MINDER Study Group, Massimo Alessandri, Fabrizio Valleggi, Giancarlo Antonucci, Sabrina Piredda, Sergio Antonio Berra, Luca Perfetti, Maria Francesca Bracale, Maurizio Burattini, Nicola Tarquinio, Giuseppe Campagna, Tommasina Giuliani, Armando Pepe, Maurizio Cavalleri, Elena Bernero, Chiara Martino, Sabrina Cerci, Antonella Leonardi, Laura Durola, Paolo Corradini, Daniela Sbocchia, Francesco D’Angelo, Tiziana La Spina, Elena Dionigi, Maria D’Avino, Giuseppe Caruso, Roberta De Angelis, Sara Rotunno, Stefano De Carli, Sabrina Spangaro, Teresiano De Franceschi, Youssef Saleh, Raffaella De Giovanni, VogrigNadia Vogrig, Giovanni Ferrari, Micaela Asti, Cirillo Nunzia, Rebasti Daniela, Anna Rita Sorrentino, Maria Ferraro, Javier Rosada, Alessio Bonaiuto, Pietro Gatti, Mariangela Barletta, Mauro Giordano, Tiziana Ciarambino, Andrea Giorgi, Margherita Guarini, Matteo Giorgi Pierfranceschi, Matteo Migazzi, Antonio Greco, Carmela Gerdano, Concetta Coronella, Martina Lombardi, Mariacarla Ventriglia, Maurizio Manini, Davide Lillo, Larizza Giovanni, Claudia Castellano, Antonino Mazzone, Noemi Brendaglia, Alberto Moggi Pignone, Antonello Ramundo, Andrea Montagnani, Michele Caselli, Ignazio Maria Morana, Angela Pastore, Maria Giuseppina Pecora, Michela Muriago, Adriana Dal Maso, Clara Pia Pafundi, Paola Novati, Daniela Cottarelli, Gabriella Oliva, Raffaella Andreozzi, Filomena Pietrantonio, Giampiero Marino, Mario Pirisi, Gian Paolo Fra, Cecilia Politi, Concetta Mancini, Marzia Turilli, Renda Maurizio, Giuseppe Miceli, Fabrizio Santoro, Martino Rosamaria, Margherita Cirillo, Maurizio Tonizzo, Ada Zanier, Cristina Trojani, Gabriella Fantini, Raffaele Zoratti, Cristina Pizzin, Petri Roberta, Girola Andrea, Luconi Maria Paola, Romanello Daniele, Bandini Giulia, Gabrielli Mariagrazia, Imburgia Mariangela, Copat Maria, Antonini Raffaella

**Affiliations:** 1 IRCCS MultiMedica, Milan, Italy; 2 Department of Medicine, Ospedale Buonconsiglio Fatebenefratelli, Naples, Italy; 3 Department of Internal Medicine, Ospedale Di Luino, Luino, Italy; 4 Department of Medicine, ULSS 7, Bassano del Grappa, Vicenza Italy; 5 Department of Internal Medicine, ASL Roma 2-Ospedale “Pertini”, Rome, Italy; 6 Internal Medicine 2-Endocrine-Metabolic Department, AULSS2 -Veneto Ospedale Di Treviso, Treviso, Italy; 7 Cardiovascular and Metabolic Medicine, AORN Ospedali Dei Colli- V. Monaldi, Naples, Italy; 8 Department of Internal Medicine, Ospedale Di Voghera, Pavia, Italy; 9 Department of Internal Medicine, Ospedale Di Colleferro, Rome, Italy; 10 Department of Internal Medicine, ASL Foggia- Ospedale Di Cerignola, Foggia, Italy; 11 Department of Internal Medicine, ASFO Di Spilimbergo, Pordenone, Italy; 12 Department of Internal Medicine, AOU Ospedale Civile Baggiovara, Modena, Italy; 13 Department of Internal Medicine, AULSS 4 Veneto Orientale- Ospedale Di Portogruaro, Venice, Italy; 14 Department of Internal Medicine, USL TOSCANA NORD OVEST- Ospedale Di Fivizzano, Massa Carrara, Italy; 15 Department of Internal Medicine, Ospedale S.S. Salvatore, Mistretta, Messina Italy; 16 Department of Advanced Medical and Surgical Sciences, University of Campania Luigi Vanvitelli, Naples, Italy; 17 Department of Internal Medicine, Ospedale Di Castelnovo ne′ Monti, Reggio Emilia, Italy; 18 Department of Internal Medicine, Ospedale Cardarelli, Naples, Italy; 19 Department of Internal Medicine, Ospedale Acquaviva Delle Fonti, Bari, Italy; 20 Department of Internal Medicine, Ospedale Di Buccheri La Ferla Fatebenefratelli, Palermo, Italy; 21 Department of Clinical Research, FADOI Foundation Study Center, Milan, Italy; 22 ASST Sette Laghi, Insubria University, Varese, Italy; 23 Department of Emergency and Internal Medicine, Isola Tiberina Hospital, Gemelli-Isola, Rom, Italy; 24 Ospedale “Sant’Andrea”, Massa Marittima (GR), Italy; 25 Ospedale “Galliera”, Genova, Italy; 26 Ospedale di Garbagnate Milanese, Milan, Italy; 27 IRCCS Istituto Nazionale di Ricovero e Cura per Anziani INRCA di Osimo, Ancona, Italy; 28 Ospedale “S. Maria Goretti”, Latina, Italy; 29 Ospedale di Chiavari, Genova, Italy; 30 Ospedale di Frascati, Rome, Italy; 31 Ospedale “F. Petruccioli”, Pitigliano (GR), Italy; 32 Ospedale di Cernusco sul Naviglio, Milan, Italy; 33 Ospedale “San Pietro Fatebenefratelli”, Rome, Italy; 34 Ospedale “Sant’Antonio” San Daniele del Friuli, Udine, Italy; 35 Ospedale “Santa Maria della Misericordia” di Albenga, Savona, Italy; 36 AUSLRomagna, Ospedale “Cervesi” di Cattolica, Rimini, Italy; 37 ASST Pavia, Ospedale di Broni e Stradella, Pavia, Italy; 38 PO Acri di Cosenza, Cosenza, Italy; 39 Ospedale di Brindisi, Brindisi, Italy; 40 P.O. Clinicizzato di Marcianise, Caserta, Italy; 41 Ospedale “San Filippo Neri”, Rome, Italy; 42 Ospedale Maggiore, Cremona, Italy; 43 IRCCS Casa Sollievo della Sofferenza di San Giovanni Rotondo, Foggia, Italy; 44 Asl Toscana Nordovest, Ospedale di Cecina, AOU Pisana, Cecina, Italy; 45 Ospedale “San Giovanni di Dio” di Orbetello, Grosseto, Italy; 46 Ospedale Civile di Legnano, Milan, Italy; 47 A.O.U. “Careggi”, Firenze, Italy; 48 Ospedale “Santa Maria della Misericordia”, Grosseto, Italy; 49 ARNAS “Garibaldi”, Catania, Italy; 50 Ospedale “Pietro Milani” Noventa Vicentina, Vicenza, Italy; 51 ASST RHODENSE, Presidio di Passirana di Rho, Milan, Italy; 52 ASL Napoli 1 Centro, Ospedale del Mare, Naples, Italy; 53 ASL Roma 6, Ospedale dei Castelli di Ariccia, Rome, Italy; 54 A.O.U. “Maggiore della Carità”, Università del Piemonte Orientale, Novara, Italy; 55 Ospedale “F. Veneziale”, Isernia, Italy; 56 Fondazione Istituto “G. Giglio” di Cefalù, Palermo, Italy; 57 Ospedale Santa Maria degli Angeli”, Pordenone, Italy; 58 Ospedale “Ceccarini” di Riccione, Rimini, Italy; 59 Ospedale di Palmanova‚ Presidio Ospedaliero Latisana-Palmanova ASUFC, Udine, Italy

**Keywords:** GLP-1RA, Glycemic control, Guidelines, SGLT-2i, Treatment adherence, Type 2 diabetes

## Abstract

Few data are available regarding the adherence to treatment guidelines in individuals with type 2 diabetes mellitus (T2DM) admitted to Internal Medicine Wards (IMW) while no information is available concerning the possible efficacy of an educational intervention aimed at improving adherence in this setting. To explore guidelines adherence and the associated impact on glycemic control in subjects with T2DM hospitalized in IMW before and after an educational intervention, we conducted a 3-phase, cluster-randomized, multicenter study. During Phase 1, we retrospectively collected data from patients with T2DM hospitalized for any cause in IMW for ≥5 days. In Phase 2, an educational training, based on the method of the educational outreach visits (EOV), was developed in 36 out of the 54 centers involved. In Phase 3, conducted 6 months after the training, we replicated the collection of data performed in Phase 1. Overall, we analyzed data from 1909 and 1662 individuals with T2DM during Phase 1 and Phase 3 of the study, respectively. No changes were observed in the difference between mean fasting glycemia levels at discharge *vs* at admission in Phase 3 comparing EOV vs NO EOV groups. A statistically significant increase in adherence to guidelines was observed from Phase 1 to Phase 3 and a trend toward higher adherence was detected when comparing the EOV and the no EOV groups. A structured educational intervention improves adherence to guidelines for managing T2DM in individuals admitted to IMW but has no effect on short-term glycemic control.

**Supplementary Information:**

The online version contains supplementary material available at 10.1007/s11739-025-04238-1.

## Introduction

Type 2 Diabetes mellitus (T2DM) is a world pandemic increasing the risk of a range of complications, especially cardiovascular. Several studies have shown that early and aggressive control of all the most relevant risk factors, in particular hyperglycemia, dyslipidemia and hypertension can effectively prevent the development of these complications [1–3].

Obtaining optimal glycemic control, stable over time, is still a difficult result to achieve. In recent years, the availability of new safe and effective drugs has significantly changed the therapeutic approach to the disease. Patients’ phenotyping, according to a range of characteristics and comorbidities, is now the recommended standard to choose the most suitable therapy [4–8].

The results of selected studies on recently introduced classes of glucose-lowering drugs, i.e. GLP1-RA and SGLT-2i, have contributed to a further development of this strategy, with positive findings in terms of reduction in cardiovascular risk, hospitalizations for heart failure, and kidney damage [4–6]. These data, together with the effects of such drugs on the risk of hypoglycemia and their role in promoting weight reduction have led international Scientific Societies to produce guidelines [7, 8] that have substantially changed the paradigms for the treatment of hyperglycemia in T2DM. These guidelines recommend the need to prioritize, for the therapeutic choice, those classes of drugs that have demonstrated a particular effect in the specific condition that characterizes the subject with T2DM (for example, a history of previous cardiovascular events, or the presence of heart failure, or obesity).

Despite the strong evidence available and the indications of the Scientific Societies, the application of these recommendations is far from being optimal [9]. Specifically, the recent changes in the therapeutic scenario appear particularly relevant for some care settings. Among them, the area of Internal Medicine in which the complexity of the patients as well as some aspects of healthcare organization (e.g., the limited duration of hospital admissions, the transition from hospital to primary care and/or outpatient clinics) make the choice and appropriateness of treatments a challenging issue. However, in this setting, the adherence to existing guidelines on the management of patients with T2DM and the possible impact of an educational intervention have been poorly explored.

To address this issue, we conducted a multicenter study to take a real-life snapshot of the management of patients with T2DM hospitalized for any cause in Italian Internal Medicine Wards (IMW) and to evaluated possible deviations between current clinical practice and the recommended guidelines (i.e. ESC-EASD and EASD-ADA) in force at the time of study start [5–8, 10]. Moreover, we evaluated the effects of a specific educational program for health care personnel in IMW, aimed at improving the management of these patients and the adherence to guidelines.

## Methods

The MINDER study (Management of patIeNts with type 2 Diabetes mEllitus hospitalized in InteRnal Medicine Units) was designed and developed by the Italian Federation of Internal Medicine Hospital Clinicians Associations (FADOI). The MINDER study was performed in 50 Italian Internal Medicine Units and carried out between September 2020–March 2023. Centers were selected according to their ability to prescribe all classes of antidiabetic drugs.

The study was conducted according to the provisions of the Helsinki Declaration. Approval was obtained from the Ethics Committees of each participating center.

It was designed as a replication of two cross-sectional surveys interspersed with an educational program, a model successfully applied by FADOI to a number of clinical conditions [11–14].

As shown in Figure S1, the study was composed by three steps. Phase 1 involved a retrospective data collection pertaining to patients ≥18 years old with diagnosis of T2DM hospitalized for any cause in IMW for ≥5 days before February 2020. Given that data collection was retrospective, patients were managed according to routine clinical practice. As per study protocol, patients in treatment with insulin or patients treated with rapid acting insulin during the hospitalization or patients hospitalized for SARS-CoV-2 infection were excluded. Patients with infectious diseases other than SARS-CoV-2 infection were included.

In Phase 2 an educational training program, defined by the study Steering Committee, and focused on current Diabetes Guidelines recommendations and on possible deviations from best clinical practice observed during Phase 1, was conducted in 36 out of the 54 participating Centers (cluster randomization, performed at the beginning of the study). The 2:1 ratio was selected in order to offer to the majority of Centers the opportunity to undergo a training program and therefore maximize the potential positive effect of the educational program on patient management. However, at the end of the study, the educational program was offered also to the remaining 18 Centers. The program was based on the method of the educational outreach visits (EOV), a 3-h face-to-face meeting between a trained diabetes specialist from outside the Center and the staff of the Center itself. This method was selected being considered as one of the most effective to modify professional practice and improve health care outcomes [15]. In addition, all members of the center staff received a distance learning program, to reinforce and deepen the contents of the outreach visit. Prior to the outreach visits, each diabetes specialist in charge of training (*n* = 9) was asked to attend a 1-day briefing workshop to gain experience with the educational program and standardize the contents to be delivered.

Phase 3 occurred approximately 6 months after the training and involved a new data collection replicating that performed in Phase 1. In both Phase 1 and Phase 3, the data collection was based on the review of the medical records of the last 40 consecutive patients (in the period December 2018–December 2020 for Phase1 and February 2022–February 2023 for Phase 3) complying with the study inclusion criteria and hospitalized in each Center.

In order to minimize the “awareness bias” (i.e. the possibility that participation in the project significantly influenced clinical attitudes in Phase 3), only one physician of the Centers of the “Control” group (not receiving EOV and distance learning) was made aware of the study design and procedures, and he/she was supported in the study data collection by nurses or by physicians who did not have the possibility of prescribing the new classes of antidiabetic drugs.

In detail, the collected information included: gender, age, anthropometric parameters, reason for hospitalization, concomitant diseases and relevant drug therapies, therapies for T2DM at admission to hospital and at discharge, laboratory results (fasting glycemia [FG] and glomerular filtration rate at hospital admission and at discharge, glycated hemoglobin) occurrence of hypoglycemia, duration and outcome of hospitalization. To improve quality of data collection, a study-specific electronic case report form (e-CRF) with central remote control was used.

For each case, an Independent Committee of Experts assessed the adherence to guidelines of antidiabetic therapy at discharge. In particular, two Experts, unaware of the study group allocation (receiving or not receiving the educational program), examined each case independently. In case of discordant evaluation, the case report was examined by a third Expert. This method and the associated specific procedures (i.e. clinical information to be analyzed, criteria for adjudication) were considered reliable and selected by the Experts prior to the beginning of the study, following an inter-rater reliability test (by Fleiss’ kappa score) performed to measure the level of agreement among multiple judges for the same item (*n* = 9). Each Expert was asked to examine 10 clinical cases and the level of agreement was measured using Fleiss’ kappa statistics. The result achieved with this assessment (repeated twice after two cycles of training) was moderate agreement (*k* < 0.5), therefore it was considered that each clinical case could not be evaluated by only one Expert.

### Sample size and statistical analysis

Sample sizes of 1440 in Test Arm (EOV GROUP) and 720 in Control Arm (NO-EOV GROUP), which were obtained by sampling 36 centers (clusters) with an average of 40 subjects each in EOV GROUP and 18 centers with an average of 40 subjects each in NOT EOV GROUP, achieve about 80% power to detect a difference between the group means of at least 10. This estimate was calculated assuming, in phase 3, a mean difference between the first and the last measurement of fasting glycemia of around 17 mg/dL in patients hospitalized in NO-EOV GROUP Centers, and of around 27 mg/dL in patients hospitalized in EOV GROUP Centers. This estimate has been done according to the previous data collected in a study performed some years ago in a very similar setting [9]. The standard deviation of subjects is about 75. The intra-cluster correlation coefficient is 0.001 (Ballpark estimate). The coefficient of variation of cluster sizes is 0.650 (Ballpark estimate). A two-sided *t* test was used with a significance level of 0.050. This test used degrees of freedom based on the number of clusters. The sample size calculations were performed using the commercial software PASS 14. According to the study design, to have two mirroring phases, in the phase 1 the sample size will be of 2160 subjects, as well as in the phase 3. All test will be two-sided and performed at the significance level of *α* = 0.05.

For primary efficacy endpoint, variation in fasting glycemia during hospitalization between the two groups of Centers (those receiving and not receiving the educational program) during phase 3 of the study, a mixed linear model with identity link function, Gaussian distribution and parameterized according to a cluster-randomized design was estimated with “treatment indicator” as fixed effect, “center indicator”, (namely the cluster randomization unit), as random effect and the dependent variable computed for each patient (subject within cluster) using the change between the first and the last measurement of fasting glycemia. Point estimate of treatment difference with associated two-sided 95% Confidence Interval was reported as least-squares estimates and computed with degrees of freedom adjusted according to Kenward–Roger method in order to take into account the cluster nature of the study. The procedure GLIMMIX of SAS Software was employed for the aforementioned analyses and computations.

Chi-square test for categorical parameters, and *t* test or Wilcoxon rank sum test, for continuous ones, were used to compare baseline characteristic and outcomes between phases and groups (phase 3 only).

The difference between variables at discharge vs. admission among the same subjects for phase 1, phase 3 EOV and phase 3 NO EOV was explored applying paired *t*-test (continuous) and McNemar test (categorical).

To outline change in the prescription’s treatment percentage changes were calculated as

$$\frac{\left(\text{number of patient with prescription at discharge}-\text{number of patient with prescription at admission}\right)}{\text{number of patient with prescription at admission}}\times 100$$

To study the adherence to guidelines of antidiabetic therapy at discharge, a multilevel logistic regression was applied with the following variables as covariates: age ($\ge$75 vs <75 years-old), presence of concomitant disease (($\ge$3 vs <3), obesity (BMI >30), previous cardiovascular event, duration of hospitalization ($\ge$7 vs <7 days), FG at admission categorized as <70 mg/dl (reference), 70–125 mg/dL, >125 mg/dL.

The demographic and clinical–pathological characteristics were appropriately summarized by means of descriptive statistics. Mean, median and related distribution parameters were reported for continuous variables, while frequencies and percentages were calculated for the different categorical variables.

## Results

In Phase 1 and in Phase 3 of the MINDER study data were collected from 1909 and 1662 patients with T2DM, respectively (total number of 3571). General characteristics of patients at baseline and at discharge are described in Table 1.

**Table 1.** General characteristics of subjects recorded at admission to IMW in Phase 1 and in Phase 3 (EOV group and NO EOV group) (Wilcoxon rank sum test)

Variable	PHASE1 (*n* = 1909)	PHASE 3		*P* value
		EOV GROUP (*n* = 1110)	NO-EOV GROUP (*n* = 552)	
Gender, *n*. (%)	1028 (53.8) M 881 (46.2) F	591 (53.2) M 519 (46.8) F	297 (53.8) M 255 (46.2)F	# 0.98 § 0.74 ¥ 0.82
Age, years mean ± SD	76.4 ± 11.2	76.4 ± 10.5	76.0 ± 11.5	# 0.38 § 0.82 ¥ 0.51
Provenance of patients, *n* (%)				
Home	1686 (88.3)	994 (89.5)	477 (86.4)	# 0.23 § 0.03 ¥ 0.002
Another dept	123 (6.4)	56 (5.0)	41 (7.4)	
Nursing home	83 (4.3)	39 (3.5)	32 (5.8)	
Long-term care facility	17 (0.9)	21 (1.9)	2 (0.4)	
BMI, *n* (%)				
Obese (>30)	405 (21.2)	248 (22.3)	112 (20.3)	# 0.58 § 0.30 ¥ 0.11
Overweight (25–30)	614 (32.2)	330 (29.7)	195 (35.3)	
Normal weight (18.5–24.9)	807 (42.3)	493 (44.4)	223 (40.4)	
Underweight (<18.4)	83 (4.3)	39 (3.5)	22 (4.0)	
Previous cardiovascular event (myocardial infarction, stroke, unstable angina, surgical revascularization), *n* (%)	779 (40.8)	418 (37.7)	245 (44.4)	# 0.13 § 0.09 ¥ 0.008
Concomitant diseases ≥3, *n* (%)	1064 (55.7)	555 (50.0)	313 (56.7)	# 0.69 § 0.002 ¥ 0.01
HbA1c (%) ± SD	7.2 ± 1.7	7.1 ± 1.3	7.2 ± 1.6	# 0.76 § 0.009 ¥ 0.12
Outcome, *n* (%)				
Discharged	1713 (89.8)	998 (90.0)	467 (84.6)	#0.007 § 0.32 ¥ 0.001
Transferred to a nursing home or other clinical facilities	78 (4.1)	40 (3.6)	38 (6.9)	
Transferred to another hospital	60 (3.1)	46 (4.1)	21 (3.8)	
Transferred to another dept	57 (3.0)	26 (2.3)	25 (4.5)	
Actions specific for T2DM at the discharge, *n* (%)				
Referral to general practitioner	672 (35.2)	369 (33.2)	213 (38.6)	#0.001 § 0.002 ¥ 0.0002
No advice	395 (20.7)	179 (16.1)	98 (17.8)	
Referral to divisional ambulatory	(25.6)	345 (31.1)	137 (24.8)	
Referral to regional diabetes specialist	313 (16.4)	194 (17.5)	76 (13.8)	
Referral to other specialist consulting	41 (2.1)	23 (2.1)	28 (5.1)	
Duration of hospitalization, median days (IQR)	9 (6–13)	9 (7–12)	10 (7–16)	# <0.0001 § 0.73 ¥ <0.0001

(#) Phase 1 vs Phase 3 no EOV; (§) Phase 1 vs Phase 3 EOV; (¥) Phase 3 no EOV vs Phase 3 EOV

*SD* Standard deviation, *IQR* Interquartile, *n* Number, *BMI* Body mass index, *HbA1c* Hemoglobin A1C

As confirmation of the complexity of patients with diabetes hospitalized in IMW, the study population had a mean age of 76 years, suffered a median of three chronic diseases, and around 40% of patients had a previous cardiovascular event.

**Fig. 1:**
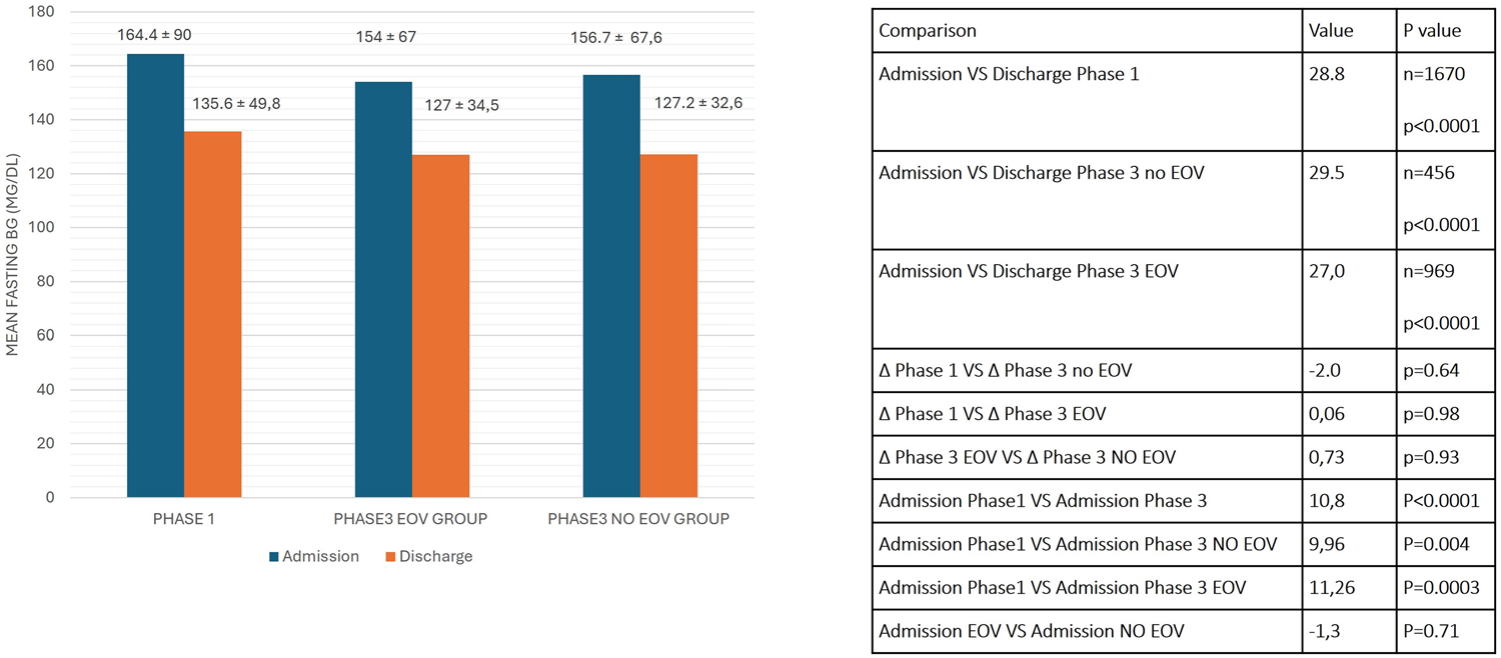
Mean fasting glycemia (FG) of patients in Phase 1 and in Phase 3 hospitalized in Centers of EOV group and NO-EOV group. Mean FG comparisons between Admission vs Discharge in Phase 1 and Phase 3 were calculated by T-test for matched data, while the variation in FG during hospitalization (∆ = Mean FG discharge – admission) between Phase 1 and Phase 3, in EOV and NO EOV group and between Phase 3 EOV and NO EOV group were calculated by Multilevel model, with center as random effect. Data relative to all the comparisons are presented in the adjacent table

In both Phase 1 and in Phase 3, mean FG at the time of hospitalization was higher than that at discharge, as a result of the clinical management during the hospitalization in IMW (Fig. 1).

We detected no significant differences for the primary endpoint of the study, i.e., the variation in FG during hospitalization between the two groups of Centers (EOV vs NO EOV) during Phase 3 of the study. The mean variation was 25.46 mg/dL in the EOV group and 26.19 mg/dL in the NO EOV group (*p* = 0.93) (Point estimate of the difference = 0.73; 95% CI −17.9;19.4) (Fig. 1).

Table 2 shows the classes of medications for the treatment of T2DM at admission and at discharge in Phase 1 and in Phase 3 for each group of Centers. In the EOV group a statistically significant higher percentage of patients received specific therapy for T2DM.

**Table 2:** Patients in therapy for T2DM in the two phases of the study

	PHASE 1 (*n* = 1909)			PHASE3 (*n* = 1662)					
	Admission	Discharge	*P* value	EOV GROUP (*n* = 1110)		*P* value	NO EOV GROUP (*n* = 552)		*P* value
				Admission	Discharge		Admission	Discharge	
Patients in therapy for T2DM *n* (%)	1559 (81.7)	1606 (84.21)	0.01	930 (83.8)	986 (88.82)	<0.0001	452 (81.9)	462 (83.7)	0.33
SGLT2i	34 (1.8)	53 (2.8)	0.008	69 (6.2)	234 (21.1)	<0.0001	15 (2.7)	58 (10.5)	<0.0001
GLP1ra	28 (1.5)	87 (4.6)	<0.0001	40 (3.6)	138 (12.4)	<0.0001	20 (3.6)	63 (11.4)	<0.0001
Metformin	1006 (52.7)	905 (47.4)	<0.0001	630 (56.8)	518 (46.7)	<0.0001	264 (47.8)	228 (41.3)	0.002
Metaglinides	191 (10.0)	150 (7.9)	0.0004	80 (7.2)	25 (2.2)	<0.0001	45 (8.1)	12 (2.2)	<0.0001
DPP4i	169 (8.8)	239 (12.5)	<00001	119 (10.7)	176 (15.9)	<0.0001	88 (15.9)	103 (18.7)	0.06
Sulfonylureas	247 (12.9)	140 (7.3)	<00001	118 (10.6)	51 (4.6)	<0.0001	48 (8.7)	19 (3.4)	<0.0001
Insulin	/	353 (18.5)	/	/	185 (16.7)	/	/	104 (18.8)	/
Combination	177 (9.3)	176 (9.2)	0.93	102 (9.2)	127 (11.4)	0.02	62 (11.2)	51 (9.2)	0.14
Other*	84 (4.4)	83 (4.3)	0.91	60 (5.4)	39 (3.5)	0.005	20 (3.6)	24 (4.3)	0.46

^*^Hypoglucidic diet, thiazolidinediones, acarbose

∆% = (discharge–admission)/admission*100 (McNemar test)

**Fig. 2:**
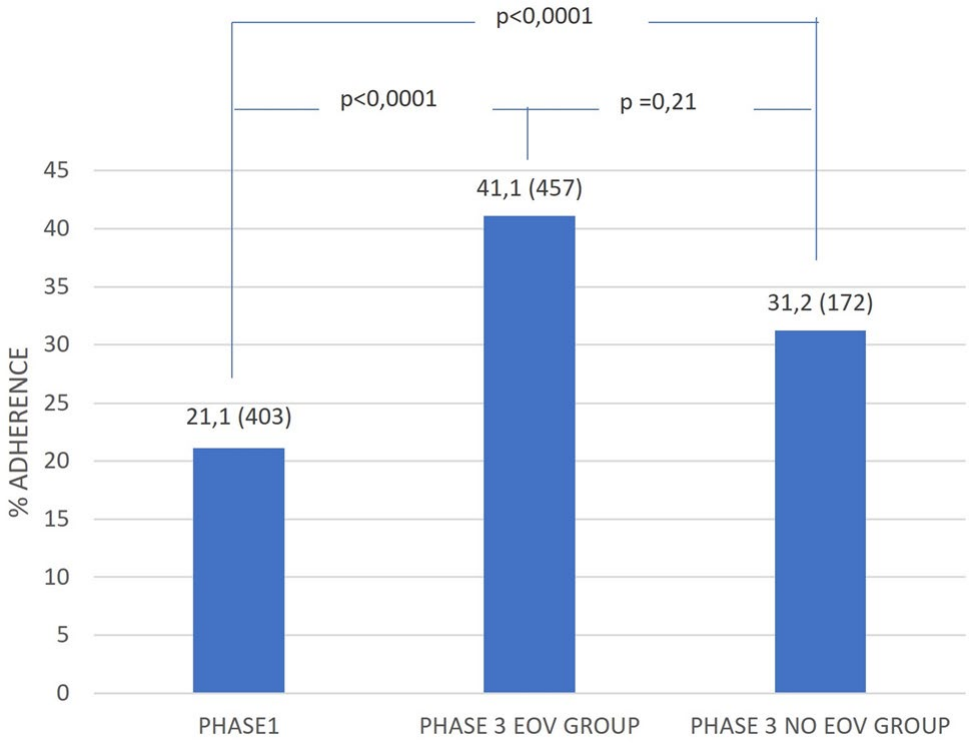
Comparison between Phase1 and Phase 3 EOV and NO EOV group in terms of percentage of adherence (n.) at T2DM therapy at the discharge (Multilevel Model)

The percentage of patients adherent to evidence-based T2DM therapy at discharge is summarized in Fig. 2. A statistically significant increase in adherence was observed from Phase1 to Phase 3 and a trend toward higher adherence was detected in the EOV group vs NO EOV.

Table 3 shows the results of univariate analyses and a multivariable logistic analysis aimed at evaluating potential predictors of non-adherence to optimal T2DM therapy in Phase 3 of the study. Previous cardiovascular events and concomitant diseases (≥3) were predictors of non-adherence in Phase1 (data not shown), but not in Phase 3 (Table 3). In Phase 3, age (≥75 years) and low FG at admission (<70 mg/dL) were associated with a significant difficulty in achieving guideline-oriented management of T2DM. On the other hand, obese patients seemed to receive a more appropriate T2DM treatment than patients with lower BMI.

**Table 3:** Univariate and multivariable multilevel logistic analysis of predictors of non-adherence in Phase 3 population

				Univariate			Multivariate		
		*N*	Not adherence	OR	IC95%	*P* value	OR	IC95%	*P* value
Age, years	<75	636	319	Reference			Reference		
	≥75	1026	714	2.1	1.6–2.6	<0.0001	2.0	1.6–2.6	<0.0001
Concomitant diseases, *n*	<3	794	469	Reference					
	≥ 3	868	564	1.1	0.9–1.4	0.31			
Obesity (BMI >30)	No	1302	834	Reference			Reference		
	Yes	360	199	0.7	0.5–0.9	0.003	0.7	0.5–0.9	0.04
Previous cardiovascular event	No	999	619	Reference					
	Yes	663	414	1.1	0.9–1.4	0.32			
Duration of hospitalization, days	<7	350	221	Reference					
	≥7	1312	812	1.0	0.7–1.3	0.88			
FG at admission, mg/dL	<70	42	35	Reference		0.009	Reference		0.04
	70–125	549	371	0.3	0.1–0.8		0.4	0.2–0.9	
	>125	1000	579	0.3	0.1–0.7		0.3	0.1–0.8	

Following the educational outreach visit, participating IMW staff (both physicians and nurses) were asked to anonymously rate their overall assessment of the event based on appropriateness of the training modality and relevant contents, clarity of exposition by the tutor, and level of interaction. The mean rating of the overall level of satisfaction with the training received and reported by the participating healthcare professionals was 8.4 (from 1 = unwelcome to 10 = welcome). In Figure S2, a qualitative stratification of the level of satisfaction for each of the four aforementioned items is shown.

## Discussion

In this 3-step study, we showed that a structured educational intervention is able to improve adherence to existing guidelines for T2DM management in patients hospitalized in IMW. However, possibly due to other educational activities on these issues occurring during the study period, even those who did not receive the educational intervention still improved their adherence, though to a lesser extent than the interventional group. Probably the well know trial effect contributed to the observed improvement also in the wards not selected for the educational program. On the other hand, it cannot be avoided that all physicians are exposed to an external communication on the guidelines, even not in a structured manner as planned in the study (e.g. participation to congresses, continuing medical education on these issues directed to internal medicine specialists).

Glycemic control improved over time, both at admission and discharge. Regarding to the main efficacy endpoint of the study, we did not detect relevant differences in glycemic control, neither between study phases nor between the EOV vs the NO EOV groups considering the ∆ admission vs discharge. Furthermore, the NO EOV group had a larger difference from that hypothesized during study design. This latter result can be attributed to an adequately established and appropriate clinical behavior for glycemic control during hospitalization. Furthermore, improvement in the management of these patients may be ascribed to the participation of clinicians in other types of educational training interventions during the period of the study. Furthermore, we must acknowledge that we did not enroll the proposed number of individuals, largely due to the impact on hospital activities driven by the Covid-19 pandemic [16], an aspect that could have affected our results.

We observed a higher use of novel glucose-lowering drugs with established cardioprotective properties after the educational program. Although this did not provide a short-term benefit on glycemic control, it is likely that the effects of increased adherence to the guidelines might provide medium or long-term benefit in terms of reduction of hard cardiovascular endpoints, which were not explored in this study. Indeed, current knowledge suggests that part of the benefit provided by both SGLT-2i and GLP-1RA is independent of their effect on glycemic control and that these drugs should be prescribed according to patient’s comorbidities and overall cardiovascular phenotype, rather than basing only on HbA1c levels [5–8, 17–21]. This aspect should be emphasized in future educational programs. Prevalent cardiovascular events and concomitant diseases (≥3) were predictors of nonadherence in Phase 1, but not in Phase 3, likely due to the effect of the educational program. Similarly, we observed a consistent increase of the percentage of referrals to divisional ambulatory and to regional diabetes specialists in the group exposed to the educational program, suggesting an improved awareness with respect to this aspect. Other predictors of nonadherence in Phase 3 were a low blood glucose level at admission and older age. Regarding the latter variable, possible influencing factors may be the range of comorbidity, overall health status, number of drugs used, and complexity of the glucose-lowering regimen, which are more common in the elderly [22].

A key aspect to consider when developing an educational program is the satisfaction of the professionals involved. The 4-item survey conducted among the staff of the IMW evidenced a high rate of satisfaction with respect to the appropriateness of the training modality, the clarity of exposition by the tutor, and level of interaction. This should reassure about the ability of the EOV approach to effectively deliver the necessary recommendation to the staff. However, given our study design, we cannot establish whether alternative methods of dissemination of guidelines, either active or passive, would have achieved similar results [23].

Beyond the difficulties encountered due to the Covid-19 pandemic and the limited sample size, which was powered to detect a difference in glycemic control but eventually not an increase in treatment adherence, this study presents additional, intrinsic limitations related to its design. First, we cannot rule out that doctors’ awareness of participating in this study might have made them more attentive to the management of diabetes, thus affecting results achieved in terms of compliance with recommendations (awareness bias). This concern would apply particularly to the results obtained during the Phase 3. However, cluster randomization of centers, receiving or not an educational program, should reassure about the reliability of the results with respect to the effect of training. Second, retrospective data collection from hospital clinical records might have missed some information required by the study protocol. On the other hand, such an approach should have limited the effect of awareness bias, providing a reliable snapshot of real-life clinical practice. Another possible limitation is that only patients not on insulin therapy were enrolled in the study, limiting the generalizability of findings. However, this choice was made with the purpose to select patients who would more plausibly benefit from an improved adherence to the guidelines. More generally, the number and geographic distribution of the IMW involved reassure about the overall representativeness of included individuals compared with the general population with T2DM. Finally, the two study groups, those exposed to the educational intervention and the control group, differed in some baseline characteristics. Unfortunately, this risk can occur with the application of the cluster randomization. This might have modestly affected the results relative to metabolic control, but they are unlikely to have influenced adherence to guidelines, as documented by the predictors of adherence emerging in Phase 3 of the study. Finally, the limited sample size did not allow subgroup analyses focusing on possible differences according to patients’ diagnosis or specific condition. In particular, the prevalence of people with infectious diseases was low and thus we could not perform a comparison between glycemia levels pre- vs post-intervention in the groups with or without infections. Given that it is common and recommended to temporarily switch to a complete insulin treatment during acute phase of infection [10], future, adequately powered studies should focus on the effect of educational programs in this specific group of individuals.

## Conclusions

In summary, our results suggest that implementing a structured educational intervention effectively enhances adherence to established guidelines for managing T2DM in individuals admitted to the IMW, even though it does not improve short-term glycemic control. Considering the well-established cardiovascular and renal benefits of novel drugs [24, 25], educational interventions aimed at improving adherence to existing guidelines might provide a long-term benefit in terms of hard outcomes for a wide population. Future, larger studies exploring this and other approaches in similar, as well as other, settings should be conducted to establish the usefulness of structured educational interventions.

## Supplementary Information

Below is the link to the electronic supplementary material.

Supplementary file1 (DOCX 233 KB)

## Data Availability

The datasets generated during and analyzed during this study are available from the corresponding author on reasonable request. The study was promoted by the FADOI Foundation’s Clinical Research Department (Fondazione FADOI - Dipartimento per la Ricerca Clinica "Centro Studi"), a not-for-profit organization which was also responsible for the scientific and operational coordination. The FADOI Foundation is also the data owner.
