# Supplementary material for: Management of subjects with type 2 diabetes hospitalized in internal medicine units: a cluster-randomized, multicenter study before and after an educational program
Source: Intern Emerg Med. 2026 Mar 13;21(3):895–905. doi: 10.1007/s11739-025-04238-1 (PMC13144241; doi:10.1007/s11739-025-04238-1)
Supplement: Supplementary file 1 — Supplementary file1 (DOCX 233 KB) [file 11739_2025_4238_MOESM1_ESM.docx]

**SUPPLEMENTARY APPENDIX**

Supplement to Antonio Ceriello et al. Management of Subjects with Type 2 Diabetes Hospitalized in Internal Medicine Units: A Cluster-Randomized, Multicenter Study Before & After an Educational Program

**Figure S1**. Flow chart of the study.


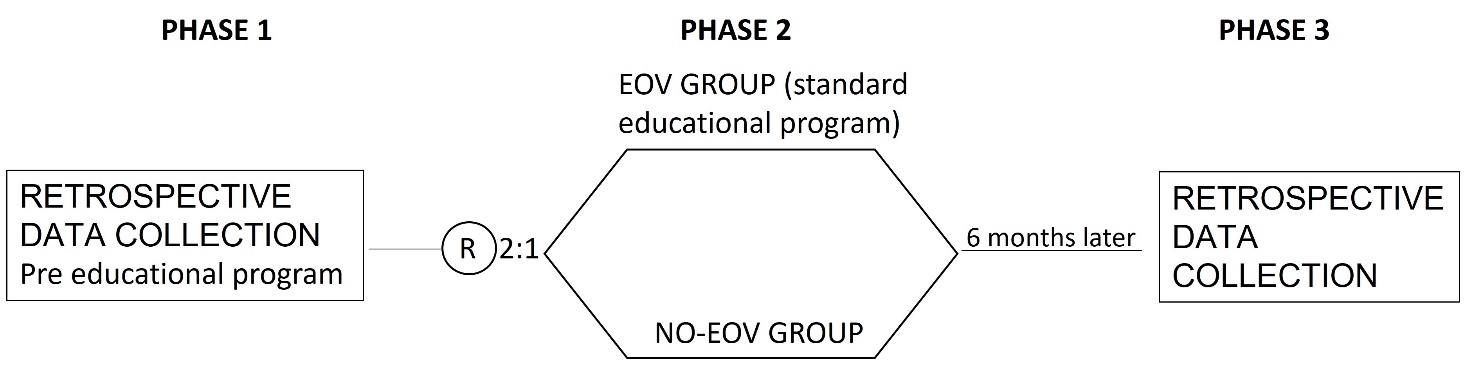


**Figure S2**. Results of the satisfaction questionnaire regarding the educational intervention completed by the participants.

**
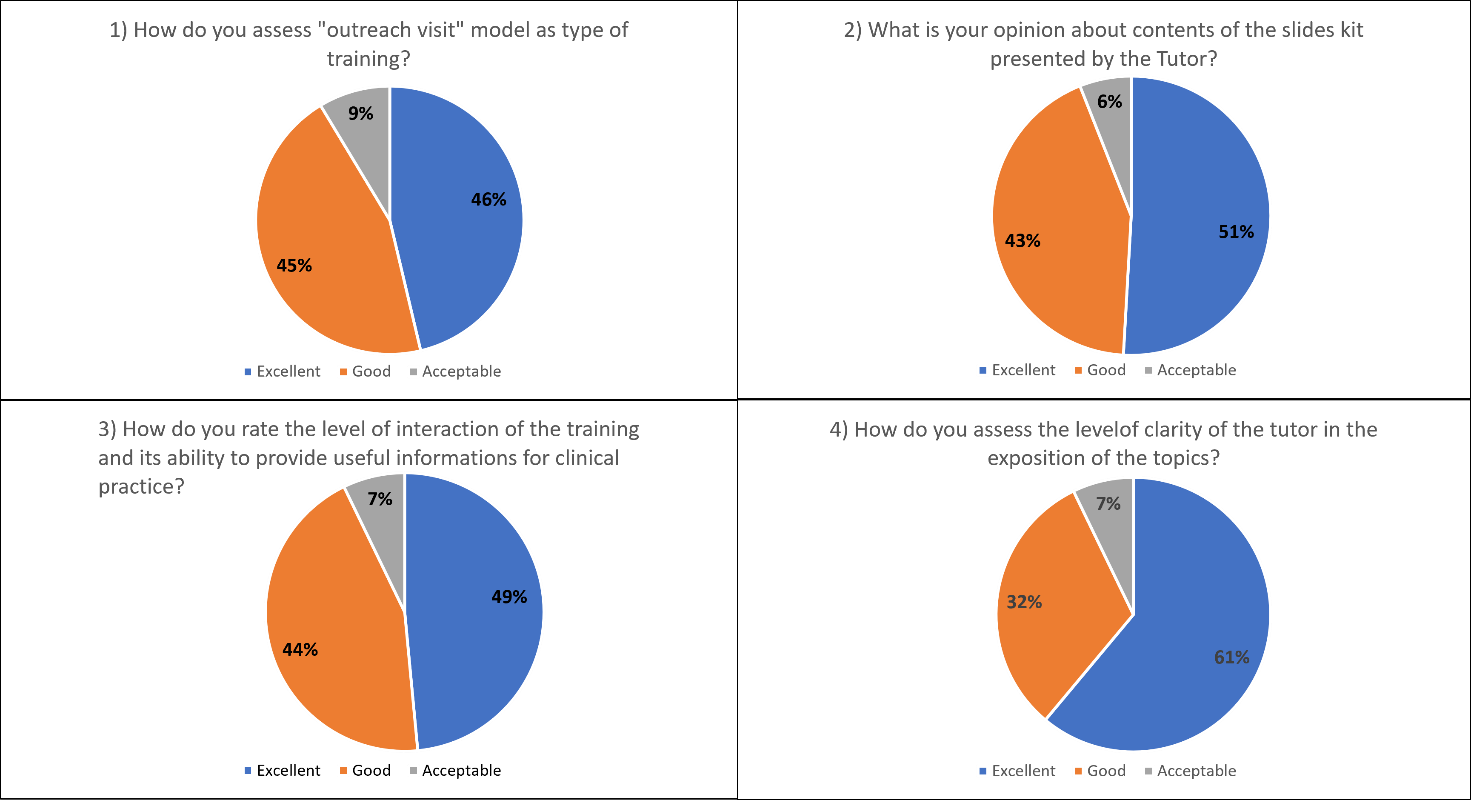
**

**Appendix**. Type of educational program used: Educational Outreach Visit (EOV)

The educational program used in the MINDER study was based on educational outreach visits (EOV) methodology.

This method was selected being considered as one of the most effective to modify health professional practice and to improve health care outcomes [14] (especially compared to educational materials or audit and feedback).

The term educational outreach is used to describe a personal visit by a trained person from outside the practice site who meets with healthcare professionals in their practice settings to provide information with the intent of changing their performance.

EOV in MINDER study was based on a 3-hour face-to-face meeting, focused on current Diabetes Guidelines recommendations, between a trained diabetes specialist, from outside the Internal Medicine Unit involved in the study, and the staff of the Center itself.

Prior to the outreach visits, each diabetes specialist in charge of training (n = 9) was asked to attend a 1-day briefing workshop to gain experience with the educational program and standardize the contents to be delivered. For this purpose, the study Steering Committee prepared one slide kid to be used by all trained specialists in each outreach visit.

The intervention also was tailored for each Center on possible deviations from the best clinical practice observed during the retrospective data collection (phase1). For this purpose, diabetes specialists showed, for each case collected for the study, the assessment on the adherence to guidelines of antidiabetic therapy at discharge performed by the Independent Committee of Experts.

The educational program of the outreach visits was based therefore on a common section on current Diabetes Guidelines recommendations and a tailored section addressed to achieve guideline-oriented management of T2DM of each Center.

In order to achieve a high level of attendance at EOV from members of the staff of the Center, all EOVs were certified as Continuing Medical Education and the IMW Director was directly involved in the organization of the meeting with the specialist. In particular, to obtain a stronger commitment all members of the staff of the IMW (both physicians and nurses) were invited personally by their Medical Director.

To reinforce and deepen the contents of the outreach visit all members of the staff of the Center received a distance learning program. Comparisons of multi-faceted interventions that included EOVs versus comparisons of EOVs alone suggests that the effect sizes of trials with multi-faceted interventions is slightly larger compared to trials in which the intervention was an EOV alone [14].

Following the educational outreach visit, participating IMW staff were asked to anonymously rate an overall assessment of the event based on appropriateness of the training modality and relevant contents, clarity of exposition by the tutor, and level of interaction.
